# Supplementary material for: COVID-Vaccines in Pregnancy: Maternal and Neonatal Response over the First 9 Months after Delivery
Source: Biomolecules. 2024 Apr 3;14(4):435. doi: 10.3390/biom14040435 (PMC11048428; doi:10.3390/biom14040435)
Supplement: Supplementary file 1 [file biomolecules-14-00435-s001.zip › biomolecules-2891150-supplementary.pdf]

	n	geometric mean (before)	sd	geometric mean (after)	sd	%	sd
Maternal RBD	32	2734.54	1.19	17114.45	1.14	+625.86	118.53

Supplementary Table S1: *Impact of booster dose on maternal antibodies: comparison between the antibody titer recorded during the follow-up checks before and after the booster.*

Supplementary Table 2: Mothers and babies demographics and clinical characteristics according COVID group.

Mothers			
	**COVID (N=34)**	**No COVID (N=64)**	**p**
**Age**			
Median [Q1, Q3]	37.6 [35.1, 39.6]	35.0 [32.6, 38.7]	0.029
**Race**			0.094*
Caucasian	34 (100%)	59 (92.2%)	
Asian	0 (0%)	1 (1.6%)	
Hispanic	0 (0%)	2 (3.1%)	
North African	0 (0%)	2 (3.1%)	
**Number of doses**			0.101
1 Dose	5 (14.7%)	19 (29.7%)	
2 Doses	29 (85.3%)	45 (70.3%)	
**GA at childbirth**			
Median [Q1, Q3]	274 [267, 282]	275 [269, 279]	0.714
**2nd dose post-partum**			
N (%)	5 (14.7%)	18 (28.1%)	0.136
**Type of delivery**			0.900
ED	26 (76.5%)	47 (73.4%)	
CS	7 (20.6%)	14 (21.9%)	
OVD	1 (2.9%)	3 (4.7%)	
**3rd dose after childbirth**			0.050
N (%)	24 (70.6%)	32 (50.0%)	
**Comorbidities**			0.533
N (%)	3 (8.8%)	10 (15.6%)	
**Maternal disease**			0.031
N (%)	1 (2.9%)	12 (18.8%)	
**Adverse Events to vaccination**			0.082
N (%)	9 (26.5%)	8 (12.5%)	

Babies			
	**COVID (N=36)**	**No COVID (N=67)**	**p**
**Sex [N, (%)]**			0.480
Female	13 (36.1%)	29 (43.3%)	
Male	23 (63.9%)	38 (56.7%)	
**Weight at birth (g)**			
Median [Q1, Q3]	3270 [2920, 3410]	3000 [2710, 3360]	0.167
**Length at birth (cm)**			
Median [Q1, Q3]	50.0 [48.0, 50.0]	49.0 [47.0, 50.0]	0.117
**Head circumference at birth (cm)**			

Median [Q1, Q3]	34.3 [33.0, 35.0]	34.0 [33.0, 34.5]	0.119
**APGAR 1’**			0.675
10	9 (25.0%)	21 (31.3%)	
9	22 (61.1%)	36 (53.7%)	
8	4 (11.1%)	7 (10.4%)	
≤7	1 (2.8%)	3 (4.5%)	
**APGAR 5’**			0.495
10	31 (86.1%)	54 (80.6%)	
9	4 (11.1%)	11 (16.4%)	
8	1 (2.8%)	2 (3.0%)	
**Feeding at T0 [N, (%)]**			0.173
Formula-fed	4 (11.1%)	11 (16.4%)	
Mixed-fed	6 (16.7%)	17 (25.4%)	
Breastfed	25 (69.4%)	30 (44.8%)	

GA = gestational age; ED: eutocic delivery; CS: Caesarean section; OVD: operative vaginal delivery.

**Caucasian vs other.*

	Vaccination Trimester						
	1st (n=11)		2nd (n=7)		3rd (n=80)		p
Mother (mean, sd)	942.82	1014.27	3207.43	2788.28	8289.94	9534.63	
(median, Q1-Q3)	807	176-1176	3015	1287-3071	4461	1955.5-10923.5	<0.001
Child (mean, sd)	1273.91	1164.23	6229.14	4215.93	9853.58	11228.87	
(median, Q1-Q3)	997	339-1810	4486	4314-6042	6103.5	2187-13563.5	0.004

Supplementary Table 3: *Mean levels of mother and child antibodies at birth according to the trimester of maternal vaccination (last dose). P value for Kruskal-Wallis test.*

	Birth		3 months		6 months		9 months	
	n=103		n=79		n=61		n=46	
Anti-S IgG Child titer AU/ml (mean, se)	3150.99	1.21	450.43	1.18	160.24	1.19	106.83	1.29
Percentage Change from birth AU/ml (mean, CI95%)			85.7	82.8-88.1	94.9	93.3-96.1	96.6	94.3-98.0

Estimated means from tobit regression

Supplementary Table 4: Mean neonatal antibody titer at birth (T0), 3 months (T1), 6 months (T2) and 9 months (T3) of life.

		Birth		3 months		6 months		9 months	
		n_{tot}=103		n_{tot}=79		n_{tot}=61		n_{tot}=46	
		mean	se	mean	se	mean	se	mean	se
No Covid	n= n_{tot}-34	2843.0	1.3	363.4	1.3	103.1	1.3	29.1	1.3
Covid	n=34	3802.7	1.4	612.4	1.2	263.5	1.2	312.0	1.3
Percentage Change from Birth									
		mean	CI95%	mean	CI95%	mean	CI95%	mean	CI95%
No Covid	n= n_{tot}-34			87.2	85.3-88.9	96.4	95.4-97.1	99.0	98.4-99.4
Covid	n=34			83.9	76.9-88.8	93.1	89.0-95.6	91.8	84.2-95.8

Estimated means from tobit regression

Supplementary Table 5: Mean neonatal antibody titer at birth (T0), 3 months (T1), 6 months (T2) and 9 months (T3) of life in the Covid Group and No- Covid Group of children.

	Birth n=47			3 months n=44			6 months n=32			9 months n=21		
	n	mean	se	n	mean	se	n	mean	se	n	mean	se
Formula fed	23	3275.3	1.6	22	416.2	1.5	17	89.5	1.6	10	24.8	1.6
Mixed fed	15	2741.0	1.7	14	356.6	1.7	10	94.4	1.7	8	21.9	1.7
Breastfed	9	3070.4	1.4	8	374.3	1.4	5	115.9	1.4	3	36.1	1.6
Percentage Change from Birth												
	n	mean	CI95%	n	mean	CI95%	n	mean	CI95%	n	mean	CI95%
Formula fed				22	87.3	83.5-90.2	17	97.3	95.8-98.2	10	99.2	99.1-99.4
Mixed fed				14	87.0	84.3-89.2	10	96.6	96.0-97.0	8	99.2	99.1-99.3
Breastfed				8	87.8	84.8-90.2	5	96.2	94.5-97.4	3	98.8	97.3-99.5

Supplementary Table 6*: Mean neonatal antibody titer at birth (T0), 3 months (T1), 6 months (T2) and 9 months (T3) of life by feeding type.*
